# Sacubitril/valsartan: research progress of multi-channel therapy for cardiorenal syndrome

**DOI:** 10.3389/fphar.2023.1167260

**Published:** 2023-05-05

**Authors:** Shuangcui Wang, Yuli Wang, Yun Deng, Jiaqi Zhang, Xijuan Jiang, Jianchun Yu, Jiali Gan, Wenyun Zeng, Maojuan Guo

**Affiliations:** ^1^ Oncology Department, First Teaching Hospital of Tianjin University of Traditional Chinese Medicine, Tianjin, China; ^2^ School of Integrative Medicine, Tianjin University of Traditional Chinese Medicine, Tianjin, China; ^3^ Traditional Chinese Medicine Department, Ganzhou People’s Hospital, Ganzhou, China

**Keywords:** cardiorenal syndrome, sacubitril/valsartan, RAAS, NPS, research progress

## Abstract

Cardiorenal syndrome (CRS) results from complex interaction between heart and kidneys, inducing simultaneous acute or chronic dysfunction of these organs. Although its incidence rate is increasing with higher mortality in patients, effective clinical treatment drugs are currently not available. The literature suggests that renin-angiotensin-aldosterone system (RAAS) and diuretic natriuretic peptide (NP) system run through CRS. Drugs only targeting the RAAS and NPs systems are not effective. Sacubitril/valsartan contains two agents (sacubitril and valsartan) that can regulate RAAS and NPs simultaneously. In the 2017 American College of Cardiology/American Heart Association/American Heart Failure (HF) ssociation (ACC/AHA/HFSA) guideline, sacubitril/valsartan was recommended as standard therapy for HF patients. The latest research shows that Combined levosimendan and Sacubitril/Valsartan markets are protected the heart and kidney against cardiovascular syndrome in rat. However, fewer studies have reported its therapeutic efficacy in CRS treatment, and their results are inconclusive. Therefore, based on RAAS and NPs as CRS biomarkers, this paper summarizes possible pathophysiological mechanisms and preliminary clinical application effects of sacubitril/valsartan in the prevention and treatment of CRS. This will provide a pharmacological justification for expanding sacubitril/valsartan use to the treatment of CRS.

## 1Introduction

In 2008, Claudio Ronco et al. (2008) defined five types of CRS based on pathophysiology, time frame, and presence or absence of cardiac and renal dysfunction. The interaction between heart and kidney function during acute and chronic dysfunction will result in a rapid deterioration in function and an increase in mortality (Ronco and Di Lullo, 2014). When acute decompensation of cardiac function leads to reduced glomerular filtration, type 1 cardiorenal syndrome occurs. Researchers have previously proposed that the decline of cardiac output and renal perfusion are the main reasons for the deterioration of renal function in type 1 and type 2 cardiorenal syndrome. However, recent studies have hypothesized that elevated central venous pressure is a more critical factor (Thind et al., 2018). When patients experience fluid overload due to worsening cardiac function, venous pressure increases and returns to the efferent arterioles; This leads to a net decrease in glomerular filtration pressure and renal injury. Other factors involved in the pathogenesis of type 1 and type 2 cardiorenal syndrome include elevated intra-abdominal pressure, activation of the renin-angiotensin-aldosterone system (RAAS), activation of sympathetic nervous syndrome, and increased renal inflammatory damage associated with heart failure (HF) (Di Lullo et al., 2017). Targeting this cycle is the main method for treating type 1 cardiorenal syndrome. Type 3 and 4 cardiorenal syndromes are more likely to be caused by volume overload caused by renal dysfunction, metabolic disorders (such as acidemia) leading to cardiac dysfunction, and neurohormonal changes associated with kidney disease. In the case of sepsis, systemic lupus erythematosus (SLE), diabetes, decompensated cirrhosis or amyloidosis, patients may develop type 5 cardiorenal syndrome; All of these diseases can lead to heart and kidney diseases (Kousa et al., 2023). Therefore, finding effective drugs for management of concomitant heart and kidney dysfunction is important.

The angiotensin receptor Recombinant Neprilysin (NEP) inhibitor sacubitril/valsartan consists of the angiotensin receptor blocker (ARB) valsartan and the NEP inhibitor (NEPI) prodrug sacubitril in a 1:1 ratio (Vardeny et al., 2013; Sible et al., 2016). PARADIGM-HF, a landmark clinical trial, found that sacubitril/valsartan is superior to angiotensin converting enzyme inhibitor (ACEI) in reducing hospitalizations and cardiovascular deaths in HF (McMurray et al., 2014; Myhre et al., 2019; Berg et al., 2020; Docherty et al., 2020). Therefore, Sacubitril/valsartan is recommended as the first choice for HF treatment with low ejection fraction (LVEF) (Vardeny et al., 2016; Böhm et al., 2017; Martens et al., 2019). To reduce the mortality and hospitalization rate of HF patients treated with ACEI or ARB alone (Di Lullo et al., 2017; Writing Committee Maddox et al., 2021). According to the results of latest clinical trials, sacubitril/valsartan is evidently curative in the treatment of CRS (Yang et al., 2019; Sabbah et al., 2020), but the specific mechanism is not well known. However, it is speculated that sacubitril/valsartan may simultaneously block RAAS and stimulate natriuretic peptide (NP) system to exert comprehensive effects, such as diuresis, natriuresis, neurohumoral imbalance regulation, excessive oxidative stress inhibition and inflammatory response reduction (Li et al., 2021). Therefore, based on RAAS and NPs, this paper reviews the possible mechanism and clinical potential of sacubitril/valsartan in CRS treatment to provide a theoretical evidence base for sacubitril/valsartan use in the treatment of CRS.

## 2 Pathophysiology of CRS

CRS is a disease that causes renal dysfunction through multiple pathophysiological mechanisms (Guazzi et al., 2013; Lin et al., 2016; Owens et al., 2017). Several hemodynamic factors, such as body fluid imbalance, inflammation and oxidative stress, contribute to the progression of CRS in acute or chronic presentation. Hemodynamics stress induces cardiac and renal perfusion insufficiency, resulting in cardiac and renal dysfunction (Gembillo et al., 2021). Patients with HF often experience a progressive deterioration in renal function due to poor renal perfusion caused by decreased cardiac output. Likewise, low positive blood flow not only reduces supraventricular tachycardia in HF patients, resulting in a sharp decline in cardiac output, but it also causes CRS by reducing perfusion pressure (Kumar et al., 2019).

Cardiorenal dysfunction is characterized by fluid imbalance, particularly volume overload. Fluid homeostasis is largely regulated by the kidneys for normal heart function (Damman et al., 2012; Levey et al., 2020). Patients with HF and end-stage renal disease, especially those not on dialysis, need to limit sodium intake to prevent deterioration of cardiac and renal function. The RAAS system is activated when cardiac output decreases due to HF. The heart, kidney, vascular wall, and other tissues typically express all components of RAAS (Ames et al., 2019). Angiotensin and other hormones in RAAS cause coronary artery vasoconstriction, vascular wall proliferation, fibrosis, cardiomyocyte hypertrophy, and interstitial fibrosis, as well as promoting collagen production and activating myocardial remodeling. When renal perfusion flow decreases, the activated RAAS induces strong vasoconstriction. In addition, aldosterone release causes water and sodium retention, aggravating renal failure (Kumar et al., 2019). Various diuretics, aldosterone antagonists, and drugs that block the renin angiotensin system are often prescribed for improved prognosis in patients with heart and kidney diseases (Cervenka et al., 2000). Besides RAAS and sympathetic nervous system (SNS), NPs can also maintain the endocrine system’s water and salt balance through its effects on heart and kidneys (Volpe, 2014).

Chronic kidney disease (CKD) and HF involve chronic inflammatory mechanism, with several pro-inflammatory biomarkers, including tumor necrosis factor-α (TNF-α), initiating spreading inflammatory cascades. In addition, TNF-α-related weak apoptosis inducers (TWEAK), interleukin-1 (IL-1) family members and interleukin-6 (IL-6) are closely associated with HF and CKD. Cell death and fibrosis are linked to these biomarkers in cardiac and renal tissue (Düsing et al., 2021).

**FIGURE 1:**
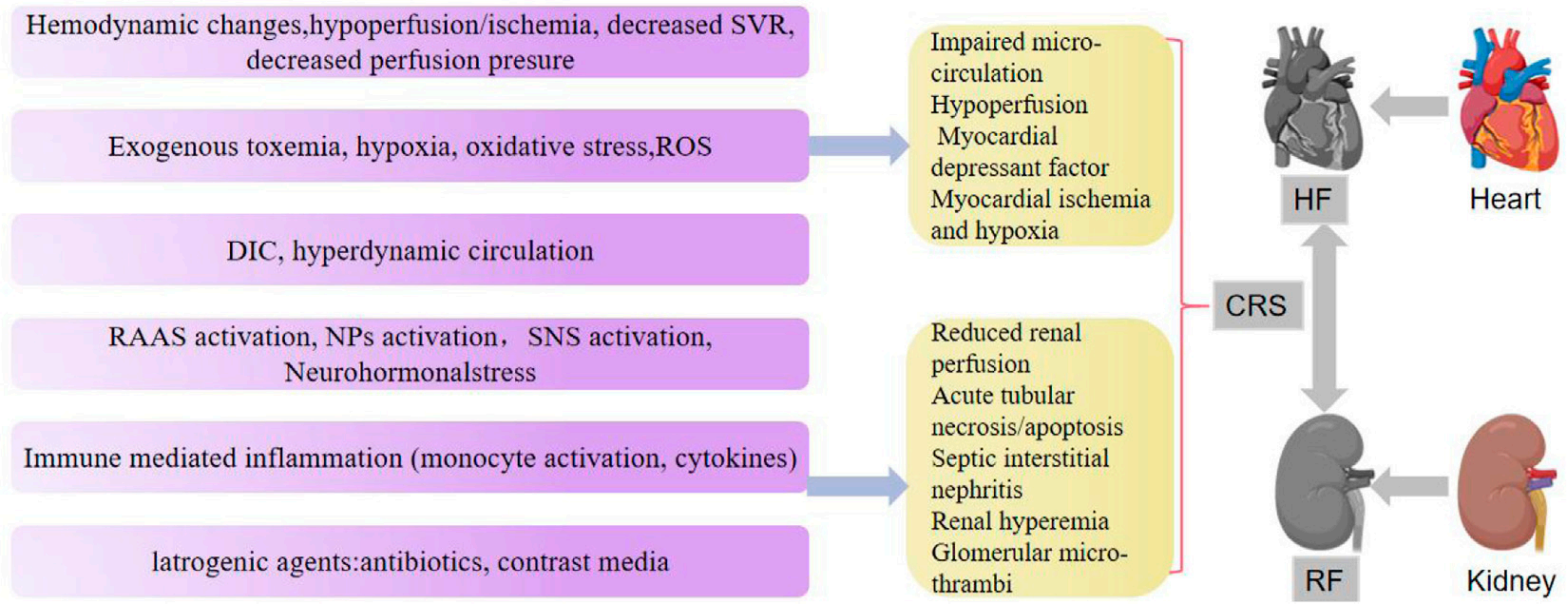
Pathophysiological and clinical features of cardiorenal syndrome.

Oxidative stress in CRS is triggered by ischemic injury, venous congestion, and inflammation. This oxidative stress is exacerbated by over-activated RAAS in patients with HF and CKD (Kumar et al., 2019). According to Virzì et al., the expression of ROS, RNs, and IL-6 was significantly increased in patients with CRS type 1 (Virzì et al., 2015). Similarly, Savira et al. (2020) found that patients with type 1 cardiorenal syndrome showed higher levels of circulating ROS than patients with acute HF alone as shown in Figure 1.

It is reported that the NPS system also plays an important role in the occurrence and development of CRS (Virzì et al., 2015). Sacubitril/valsartan can simultaneously regulate RAAS and NPs systems. Early combinations of NEP inhibitor and ACEI further increased the level of bradykinin, which significantly increased edema. Compared with ACEI, ARB does not alter bradykinin metabolism, which is thought to cause vasoedema associated with Visceral pleural infiltration (VPI). Therefore, it makes more sense to combine NEP inhibitors with ARBs (Yuan et al., 2010; Domenig et al., 2016; Sankhe et al., 2021). While NEP inhibition can regulate natriuretic and aldosterone, its benefit to hemodynamics is not entirely understood (Chen and Burnett, 2017).

SVR, systemic vascular resistance; ROS, reactive oxygen species; DIC, disseminated intravascular coagulation; SNS, sympathetic nervous system; RAAS, renin-angiotensin-aldosterone system; NPs, natriuretic peptides.

## 3 Analysis of mechanism of multi-target prevention and treatment of CRS by sacubitril/valsartan from its components

“Sacubitril/Valsartan” is a salt complex of sacubitril and valsartan in a 1:1 ratio. This compound inhibits RAAS and activates the NPs system. Research has established the role of RAAS in heart and kidney disease pathophysiology (Ziff et al., 2016). Recent studies have shown that NPs also play a role in heart and kidney diseases (Volpe, 2014).

### 3.1 Sacubitril/valsartan activates NPs system to improve CRS

Atrial type NPs (ANPs), brain type NPs (BNPs), and C type NPs (CNPs) are the most common NPs (Potter et al., 2009; Abuzaanona and Lanfear, 2017). Cardiac hormones ANP and BNP are synthesized and secreted by atrial and ventricular myocytes, respectively (Nakagawa et al., 2019). NPs perform numerous physiological functions, including inhibiting the RAAS system and endothelin expression and stimulating vasodilation (Bayes-Genis et al., 2016). Sacubitril is a prodrug, that is, rapidly metabolized into active NEPI (NEP inhibitor). By reducing degradation of NPs, sacubitril increases the concentration of NPs. It also stimulates synthesis of cyclic guanosine phosphate (CGMP) by connecting receptors to guanylcyclase. cGMP increases glomerular hemodynamics, which causes a decrease in sodium reabsorption in the kidneys, facilitates vasodilation of afferent arterioles and promotes myocardial relaxation. It also increases renal blood flow and glomerular filtration rate (D'Elia et al., 2017). This expands blood vessels, reduces blood pressure, and improves ventricular remodeling (Oatmen et al., 2018). NEPI has promising benefits to patients with heart and kidney disease, and is now an optimal treatment option for CRS.

### 3.2 Sacubitril/valsartan inhibits RAAS and improves heart and kidney diseases

RAAS regulates cardiovascular, renal, and adrenal functions, improving fluid, electrolyte, and arterial pressure homeostasis (Ames et al., 2019). The classic RAAS consists of the circulatory and endocrine systems. Angiotensin II (Ang II) is the principal effector hormone resulting from renin-mediated conversion of angiotensinogen to its precursor angiotensin I (Ang I) in the first and speed-limiting step of RAAS (Romero et al., 2015). RAAS activation typically serves as the initial compensatory response to hypoperfusion (such as early heart disease and kidney disease), but its continual activation contributes to the development of HF and kidney disease (Singhania et al., 2020). Ang I, Ang II, bradykinin (BK), endothelin-1 (ET-1) and other vasoactive substances can be degraded by NEP (neutral endorphinase). NEP, for example, can hydrolyze AngⅠ to angiotensin I-VII, activate ET-1, and catalyze BK (Chinese) to inactive BK1-7 (Sankhe et al., 2021). In addition to increasing the level of NPs, neutral endorphinase inhibitor (NEPI) also increased.

The concentration of Ang II in the circulation, offsetting the positive effects of NPs (Singh et al., 2017). According to studies, inhibiting NEP alone has a greater vasoconstrictory effect than vasodilation alone (Oatmen et al., 2018). Therefore, NEPI alone can activate RAAS and cause kidney disease.

NEPI is only effective when combined with RAAS blockers; otherwise, activating RAAS will worsen CKD (Vejakama et al., 2017). NEPI and ACE further increased bradykinin, increasing the risk of edema (Haynes et al., 2020). Therefore, this effect gives a pharmacological justification for agents combining NEPI and ARB. Valsartan is an angiotensin receptor blocker (ARB), which can significantly lower levels of AngII *in vivo*. Sacubitril/valsartan inhibits the binding of Ang II to its receptor by binding NEPI and ARB. Therefore, the combination is designed provide cardiorenal protection (Haynes et al., 2020).

Additionally, NEP can catalyze opioid peptides, such as substance P, involved in inflammation regulation β-amyloid, and gastrin. Notably, selective inhibition of NEP also produces broader effects than expected, hence it merits further investigation in clinical trials (Chen and Burnett, 2017).

## 4 Multichannel therapy with sacubitril/valsartan for cardiorenal diseases

### 4.1 Sacubitril/valsartan treats heart and kidney diseases by improving body fluid imbalance

Based on the regulation of NPs and RAAS, this paper discusses three possible mechanisms of action of sacubitril/valsartan in the treatment of CRS: body fluid imbalance, oxidative stress and inflammation. Clinical symptoms of cardiac and renal dysfunction are caused by fluid imbalance (Cervenka et al., 2000), which also increases the risk of renal syndrome. Whether the body fluid imbalance is caused by systemic congestion due to HF or sodium water retention in renal failure, it can activate NPs by inhibiting NEP. On the one hand, NPs act on the heart and kidneys and maintains the balance of water and salt and the internal environment. Additionally, they can avoid the retention of water and sodium in patients with CHF, delaying the process of cardiac decompensation (Mueller et al., 2019). When kidneys inhibit NPs secretion, NPs gradually lose their natriuretic effect and less sodium is excreted into urine. Debold et al. confirmed that the atrium can reduce blood pressure by stimulating sodium excretion and drainage from the kidneys, indicating that in addition to maintaining water and sodium balance, the heart is an endocrine organ (de Bold et al., 1981). Therefore, sacubitril/valsartan can simultaneously regulate humoral metabolism of both the heart and kidney through NPs. NEPI, which also inhibits Ang I, can improve the water sodium content and volume balance in CKD patients. RAAS inhibition by NPs induces natriuretic and diuretic effects of NEPI, increasing sodium bioavailability and reducing blood pressure, (Polhemus et al., 2017; Domondon et al., 2019). Through RAAS, cardiorenal protection can be blocked. ARB in combination with angiotensin type 1 receptor (AT1) prevents vasoconstriction and reduces sodium retention and water absorption (Ruiz-Hurtado and Ruilope, 2015). Martin et al. (2005) found that inhibiting NEP may have potential therapeutic value in the treatment of comorbid kidney disease and progressive HF. In the study by Polina et al. (2020), proteinuria was mildly relieved only in rats treated with sacubitril/valsartan. Therefore, sacubitril/valsartan can improve humoral imbalance and treat CRS through RAAS and NPs.

### 4.2 Sacubitril/valsartan treats heart and kidney diseases by improving inflammatory response

Chronic inflammation is present in both CKD and HF (Brown, 2013; Chen et al., 2017). Pro-inflammatory biological factors, such as IL-6, TNF-a, and COX-2, contribute to the development of these diseases by recruiting various inflammatory factors to the site of injury (Pacurari et al., 2014; Muñoz-Durango et al., 2016) and aggravating inflammatory response. Recent studies have shown that inflammation and NPs are closely associated (Fish-Trotter et al., 2020). NPR (NP receptor)-1 has been found to participate in immune and inflammatory responses (Zhang et al., 2015). Experimental results have shown that NRP-1 knockout mice have activated nuclear factor kappa B (NF-κB) in their kidneys and hearts (Vellaichamy et al., 2005; Das et al., 2012), indicating that NPs have anti-inflammatory properties. Studies have shown that sacubitril/valsartan inhibits continuous phosphorylation of JNK, p38MAPK, and NF-κ and nuclear translocation of B produces anti-inflammatory effects in the cardiovascular system (Ge et al., 2019). Additionally, RAAS plays an important role in the regulation of cardionephritis (Simões E Silva and Teixeira, 2016). In the treatment of heart and kidney diseases, sacubitril/valsartan can also improve the inflammatory response by blocking RAAS (Simões E Silva and Teixeira, 2016).

### 4.3 Sacubitril/valsartan improves oxidative stress in the treatment of heart and kidney diseases

**FIGURE 2:**
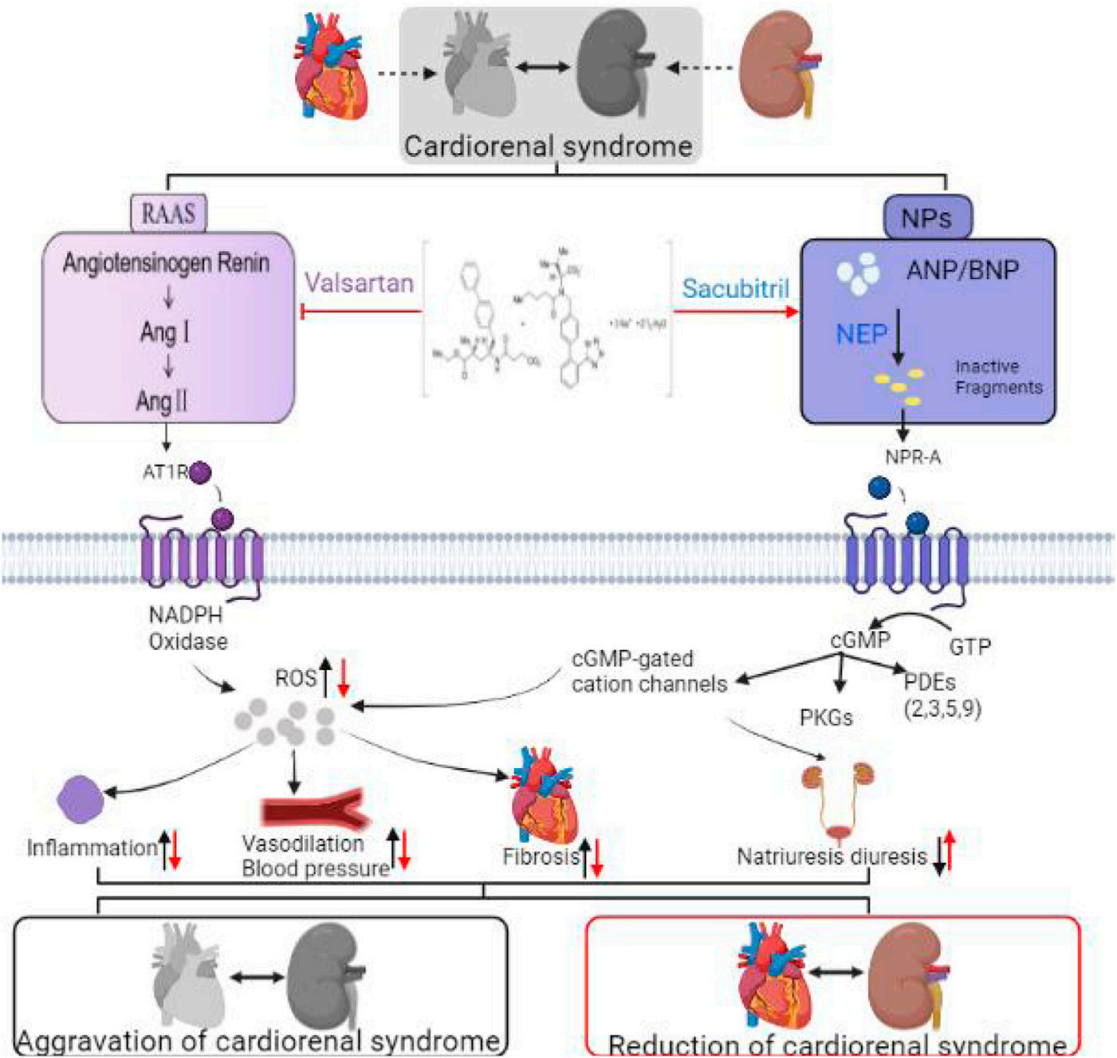
Mechanism of sacubitril/valsartan in the treatment of cardiorenal syndrome.

Cell damage is caused by oxidative stress, a common pathological mechanism in the development of chronic disease (Virzì et al., 2015). In the CRS environment, oxidative stress can be triggered by ischemic injury, venous congestion (which leads to periendothelial cell membrane stress), and inflammation. Studies have shown that ANP inhibits ROS production by reducing NADPH oxidase, which is the principal source of ROS in the heart (Laskowski et al., 2006). Researchers found that sacubitril/valsartan upregulated MnSOD and SIRT3 (Suematsu et al., 2018; Peng et al., 2020) and reduced ROS (Ge et al., 2019), improving the oxidative stress response. In previous studies, it was demonstrated that the bioavailability of nitric oxide (NO) decreased in HF with preserved LVEF (HFPEF) animals and patients. This supports the hypothesis that cGMP PKG signal transduction may be damaged in HFPEF due to high oxidative stress, leading to low myocardial NO bioavailability (Franssen et al., 2016). Sacubitril/valsartan may exert a protective effect on cardiac and vascular functions by increasing the bioavailability of NO. High levels of NO can alleviate HF symptoms and signs through angiogenesis and vasodilation, as well as change the pathological progress of HF by altering oxidative stress and hypertrophy as shown in Figure 2; (Trivedi et al., 2018).

By promoting the NPs system and inhibiting the RAAS system, sacubitril/valsartan induces pluripotent biological effects. NPs activate NPR-A receptors that produce cGMP, which binds to protein kinase G (PKG), ion channels, and phosphodiesterases (PDEs). Although NP clearance receptors (NPRCs) lack guanylyl cyclase activity, they can mediate NP endocytosis. At the same time, together with the inhibited RAAS system, it reduced inflammation and vasodilation pressure, improved fibrosis, and promoted diuresis and sodium excretion.

## 5 Multidimensional clinical evaluation of the therapeutic effect of sacubitril/valsartan on cardiorenal syndrome

### 5.1 Efficacy of sacubitril/valsartan in treatment of CRS with heart failure and nephropathy based on glomerular filtration rate

An estimated glomerular filtration rate (EGFR) is used to measure renal function and hemodynamic changes. HF and CKD share many risk factors. Therefore, CKD is likely to be more prevalent in HF patients. About half of patients with HF also have CKD, increasing their risk of death and morbidity (Vindhyal et al., 2018). Studies have shown that sacubitril/valsartan improves cardiac and renal function in patients with HF and renal dysfunction (Voors et al., 2015; Damman et al., 2018; Packer et al., 2018; Chang et al., 2019; Spannella et al., 2019; Mc et al., 2020). The PARADIGM-HF trial prospectively compared angiotensin receptor-neprilysin inhibitor (ARNI) and ACE inhibition to determine their impact on overall mortality and incidence rate of HF, consistent with the concept of CRS described by Ronco et al.

In a study of the effect of sacubitril/valsartan on the decline of EGFR, 8399 HF patients with decreased LVEF were treated with sacubitril/valsartan or enalapril. Compared with enalapril, sacubitril/valsartan treatment showed an improvement in EGFR levels and heart function (Damman et al., 2018), suggesting that in HF patients, sacubitril/valsartan reduced renal dysfunction. Packer et al. (2018), randomized, double-blind PARADIGM-HF trial compared the efficacy of sacubitril/valsartan (97 mg/103 mg, twice daily) and enalapril (10 mg, twice daily) in 8399 patients with mild to moderate chronic heart disease. The decline rate of renal EGFR was lower in patients treated with sacubitril/valsartan than in those treated with enalapril.

Sacubitril/valsartan slows down the decline of EGFR consistently across time points. Causland et al. found that sacubitril/valsartan reduced the risk of renal events and slowed the decline of EGFR in HF patients with HFPEF (Spannella et al., 2019). In their trial, Voors et al. assigned 301 HFPEF patients randomly to sacubitril/valsartan or valsartan. In this study, EGFR decrease was lower in the sacubitril/valsartan group than in the valsartan group. Regardless of time point, the incidence of worsening renal function (WRF) in the sacubitril/valsartan group (12%) was lower than that in the valsartan-only group (18%) (Chang et al., 2019).

Sacubitril/valsartan slows down the decline of EGFR, with the most benefitting age being under 65. Spannella et al. observed and analyzed 54 consecutive HF outpatients with a decrease in LVEF and clinical indications for sacubitril and valsartan. During the follow-up period, the systolic blood pressure (BP) decreased, whereas the left ventricular ejection fraction (LVEF) only increased slightly. In addition, renal function improved after 12 months compared with the historical control group, with subjects younger than 65 years old and those with CKD benefitting the most (Mc et al., 2020).

Sacubitril/valsartan has been included in HF prevention and treatment guidelines is an effective and safe pharmacological therapy for use. In Chang et al.‘s study, 466 HF with reduced LVEF (HFrEF) patients received sacubitril/valsartan treatment (group A) and 466 patients received standard HF treatment without ARNI (group B) in HF referral center. Sacubitril/valsartan appeared to be effective treatment for HFrEF patients, including those with late renal function impairment caused by CRS (Voors et al., 2015).

### 5.2 Treatment of CRS with renal failure and heart failure with sacubitril/valsartan based on cardiac markers

Cardiac markers not only indicate cardiac function, but also renal function. Acute heart failure (AHF) is closely associated with changes in renal function (usually assessed by changes in creatinine or EGFR). In about 30%–50% of patients hospitalized with AHF, renal function declines or improves during their hospital stay (Mullens et al., 2020). Clinical studies have shown that sacubitril/valsartan can simultaneously treat renal insufficiency and HF. In Heyse et al. (2010) study, a 67-year-old man with ischemic cardiomyopathy and renal insufficiency received hemodialysis for HF with HFrEF. The NT-proBNP level decreased significantly and the filling pressure decreased after the patient started receiving sacubitril/valsartan. In a study by Haynes et al., sacubitril/valsartan reduced the average systolic and diastolic blood pressure by 5.4 (95% CI, 3.4-7.4) and 2.1 (95% CI, 1.0-3.3) mm Hg, respectively. Additionally, troponin I and N levels of the hormone pro-brain NP terminal (tertiary endpoint) increased by 16% (95% Ci, 8-23) and 18% (95% Ci, 11-25), respectively. The effect of sacubitril/valsartan on renal function and proteinuria was similar to that of irbesartan after 12 months. However, in patients with chronic kidney disease, it also reduced blood pressure and cardiac biomarkers (Haynes et al., 2018).

Presently, there is no standard therapeutic drug for CRS due to its complex pathogenesis. The first angiotensin receptor NEP inhibitor to be approved is sacubitril/valsartan, which is composed of two drugs, the ARB valsartan and the NEP inhibitor prodrug sacubitril. Both NPs and RAAS can be inhibited by sacubitril/valsartan in the heart and kidneys. Considering the complex pathogenesis of CRS, sacubitril/valsartan exerts protective effects in various ways. Animal experiments and clinical studies have demonstrated that sacubitril/valsartan can reduce the risk of cardiovascular disease and delay deterioration in renal function in people with CRS (Table 1). Related studies have shown that Combined levosimendan and Sac/Val were superior to merely one another on protecting the heart and kidney as well as preserving their functions against double ischemia -reperfusion (IR) injury. As the drug is increasingly being applied in clinical practice, its pharmacological mechanisms need to be explored to improve its safety. In conclusion, NEP may be an important component of sacubitril/valsartan in the treatment of heart and kidney disease. However, more clinical and basic studies involving CRS patients alone are warranted in the future to determine the potential efficacy and safety of sacubitril/valsartan in these patients.

**TABLE 1:** Clinical application of sacubitril/valsartan in the treatment of heart and kidney diseases.

Disease	Evaluation index	Other evaluation indicators	Patient	Conclusion	Reference
HEART FAILURE WITH NEPHROPATHY	Glomerular filtration rate (EGFR)	UACR	Sacubitril/valsartan or enalapril were randomized to 8399 HF patients with HFrEF	Reduce EGFR and improve cardiac blood circulation	Vindhyal et al. (2018)
			8399 patients with mild to moderate chronic HF and systolic dysfunction were compared	EGFR descent speed is slower	Damman et al. (2018)
		Glomerular filtration rate (EGFR)	HF patients with HFPEF received sacubitril/valsartan (n = 2419) or valsartan (n = 2403)	Reduces the risk of renal events in HF patients with HFPEF and slows the decline of EGFR	Packer et al. (2018)
		Creatinine, Cystatin C and urinary albumin to creatinine ratio (UACR)]	301 HFPEF patients were randomly assigned into sacubitril/valsartan or valsartan group	EGFR decreases and UACR increases in HFPEF patients	Spannella et al. (2019)
	LVEF decrease	Systolic blood pressure (BP), left ventricular LVEF (LVEF)	54 consecutive outpatients with decreased LVEF and sacubitril/valsartan clinical indications	The systolic blood pressure decreases, the left ventricular ejection fraction slightly increases, and the improvement of EGFR is significant	Chang et al. (2019)
		Baseline	466 HFREF patients were treated with sacubitril/valsartan, whereas 466 other patients were treated with standard HF without ARNI	Effectiveness in HFREF patients, including patients with advanced renal damage	Mc et al. (2020)
Renal insufficiency leads to HF	NT-proBNP	Echocardiography	Due to ischemic cardiomyopathy and renal insufficiency	The filling pressure decreases and NT-proBNP significantly decreases	Mullens et al. (2020)
		Creatinine, UACR and cardiac biomarkers	Salkubil/valsartan and irbesartan were given to 207 subjects	Impacts on blood pressure and cardiac biomarkers	Heyse et al. (2019)

## References

[B1] Abuzaanona A. Lanfear D. (2017). Pharmacogenomics of the natriuretic peptide system in heart failure. Curr. heart Fail. Rep. 14 (6), 536–542. 10.1007/s11897-017-0365-5 29075957 PMC5681877

[B2] Ames M. K. Atkins C. E. Pitt B. (2019). The renin-angiotensin-aldosterone system and its suppression. J. Vet. Intern Med. 33 (2), 363–382. 10.1111/jvim.15454 30806496 PMC6430926

[B3] Bayes-Genis A. Barallat J. Richards A. M. (2016). A test in context: Neprilysin: Function, inhibition, and biomarker. J. Am. Coll. Cardiol. 68 (6), 639–653. 10.1016/j.jacc.2016.04.060 27491909

[B4] Berg D. D. Braunwald E. DeVore A. D. Lala A. Pinney S. P. Duffy C. I. (2020). Efficacy and safety of sacubitril/valsartan by dose level achieved in the PIONEER-HF trial. JACC. Heart Fail. 8 (10), 834–843. 10.1016/j.jchf.2020.06.008 32800511 PMC7541586

[B5] Böhm M. Young R. Jhund P. S. Solomon S. D. Gong J. Lefkowitz M. P. (2017). Systolic blood pressure, cardiovascular outcomes and efficacy and safety of sacubitril/valsartan (LCZ696) in patients with chronic heart failure and reduced ejection fraction: Results from PARADIGM-HF. Eur. heart J. 38 (15), 1132–1143. 10.1093/eurheartj/ehw570 28158398 PMC6251522

[B6] Brown N. J. (2013). Contribution of aldosterone to cardiovascular and renal inflammation and fibrosis. Nat. Rev. Nephrol. 9 (8), 459–469. 10.1038/nrneph.2013.110 23774812 PMC3922409

[B7] Cervenka L. Símová M. Malý J. Heller J. (2000). Role of the kidney in long-term regulation of blood pressure and the development of hypertension. Ceskoslovenska Fysiol. 49 (3), 116–133. 11039242

[B8] Chang H. Y. Feng A. N. Fong M. C. Hsueh C. W. Lai W. T. Huang K. C. (2019). Sacubitril/valsartan in heart failure with reduced ejection fraction patients: Real world experience on advanced chronic kidney disease, hypotension, and dose escalation. J. Cardiol. 74 (4), 372–380. 10.1016/j.jjcc.2019.03.010 30982680

[B9] Chen L. Deng H. Cui H. Fang J. Zuo Z. Deng J. (2017). Inflammatory responses and inflammation-associated diseases in organs. Oncotarget 9 (6), 7204–7218. 10.18632/oncotarget.23208 29467962 PMC5805548

[B10] Chen Y. Burnett J. J. (2017). Biochemistry, therapeutics, and biomarker implications of neprilysin in cardiorenal disease. Clin. Chem. 63 (1), 108–115. 10.1373/clinchem.2016.262907 28062615 PMC6613397

[B11] D'Elia E. Iacovoni A. Vaduganathan M. Lorini F. L. Perlini S. Senni M. (2017). Neprilysin inhibition in heart failure: Mechanisms and substrates beyond modulating natriuretic peptides. Eur. J. Heart Fail 19 (6), 710–717. 10.1002/ejhf.799 28326642

[B12] Damman K. Gori M. Claggett B. Jhund P. S. Senni M. Lefkowitz M. P. (2018). Renal effects and associated outcomes during angiotensin-neprilysin inhibition in heart failure. JACC Heart Fail 6 (6), 489–498. 10.1016/j.jchf.2018.02.004 29655829

[B13] Damman K. Voors A. A. Navis G. van Veldhuisen D. J. Hillege H. L. (2012). Current and novel renal biomarkers in heart failure. Heart Fail Rev. 17 (2), 241–250. 10.1007/s10741-011-9254-2 21604178 PMC3310988

[B14] Das S. Periyasamy R. Pandey K. N. (2012). Activation of IKK/NF-κB provokes renal inflammatory responses in guanylyl cyclase/natriuretic peptide receptor-A gene-knockout mice. Physiol. Genomics 44 (7), 430–442. 10.1152/physiolgenomics.00147.2011 22318993 PMC3339852

[B15] de Bold A. J. Borenstein H. B. Veress A. T. Sonnenberg H. (1981). A rapid and potent natriuretic response to intravenous injection of atrial myocardial extract in rats. Life Sci. 28 (1), 89–94. 10.1016/0024-3205(81)90370-2 7219045

[B16] Di Lullo L. Bellasi A. Barbera V. Russo D. Russo L. Di Iorio B. (2017). Pathophysiology of the cardio-renal syndromes types 1-5: An uptodate. Indian Heart J. 69 (2), 255–265. 10.1016/j.ihj.2017.01.005 28460776 PMC5415026

[B17] Docherty K. F. Vaduganathan M. Solomon S. D. McMurray J. (2020). Sacubitril/Valsartan: Neprilysin inhibition 5 Years after PARADIGM-HF. JACC. Heart Fail. 8 (10), 800–810. 10.1016/j.jchf.2020.06.020 33004114 PMC8837825

[B18] Domenig O. Manzel A. Grobe N. Königshausen E. Kaltenecker C. C. Kovarik J. J. (2016). Neprilysin is a mediator of alternative renin-angiotensin-system activation in the murine and human kidney. Sci. Rep. 6, 33678. 10.1038/srep33678 27649628 PMC5030486

[B19] Domondon M. Nikiforova A. B. DeLeon-Pennell K. Y. Ilatovskaya D. V. (2019). Regulation of mitochondria function by natriuretic peptides. Am. J. physiology. Ren. physiology 317 (5), F1164–F1168. 10.1152/ajprenal.00384.2019 PMC6879937 31509010

[B20] Düsing P. Zietzer A. Goody P. R. Hosen M. R. Kurts C. Nickenig G. (2021). Vascular pathologies in chronic kidney disease: Pathophysiological mechanisms and novel therapeutic approaches. J. Mol. Med. (Berlin, Ger. 99 (3), 335–348. 10.1007/s00109-021-02037-7 PMC7900031 33481059

[B21] Fish-Trotter H. Ferguson J. F. Patel N. Arora P. Allen N. B. Bachmann K. N. (2020). Inflammation and circulating natriuretic peptide levels. Circ. Heart Fail 13 (7), e006570. 10.1161/CIRCHEARTFAILURE.119.006570 32507024 PMC7375923

[B22] Franssen C. Chen S. Unger A. Korkmaz H. I. De Keulenaer G. W. Tschöpe C. (2016). Myocardial microvascular inflammatory endo-thelial activation in heart failure with preserved ejection fraction. JACC Heart Fail. 4, 312–324. 10.1016/j.jchf.2015.10.007 26682792

[B23] Ge Q. Zhao L. Ren X. M. Ye P. Hu Z. Y. (2019). LCZ696, an angiotensin receptor-neprilysin inhibitor, ameliorates diabetic cardiomyopathy by inhibiting inflammation, oxidative stress and apoptosis. Exp. Biol. Med. (Maywood) 244 (12), 1028–1039. 10.1177/1535370219861283 31262190 PMC6879777

[B24] Gembillo G. Visconti L. Giusti M. A. Siligato R. Gallo A. Santoro D. (2021). Cardiorenal syndrome: New pathways and novel biomarkers. Biomolecules 11 (11), 1581. 10.3390/biom11111581 34827580 PMC8615764

[B25] Guazzi M. Gatto P. Giusti G. Pizzamiglio F. Previtali I. Vignati C. (2013). Pathophysiology of cardiorenal syndrome in decompensated heart failure: Role of lung-right heart-kidney interaction. Int. J. Cardiol. 169 (6), 379–384. 10.1016/j.ijcard.2013.09.014 24182905

[B26] Haynes R. Judge P. K. Staplin N. Herrington W. G. Storey B. C. Bethel A. (2018). Effects of sacubitril/valsartan versus irbesartan in patients with chronic kidney disease. Circulation 138 (15), 1505–1514. 10.1161/CIRCULATIONAHA.118.034818 30002098

[B27] Haynes R. Zhu D. Judge P. K. Herrington W. G. Kalra P. A. Baigent C. (2020). Chronic kidney disease, heart failure and neprilysin inhibition. Nephrol. Dial. Transpl. 35 (4), 558–564. 10.1093/ndt/gfz058 PMC7139204 31028383

[B28] Heyse A. Manhaeghe L. Mahieu E. Vanfraechem C. Van Durme F. (2019). Sacubitril/valsartan in heart failure and end-stage renal insufficiency. Esc. Heart Fail 6 (6), 1331–1333. 10.1002/ehf2.12544 31668014 PMC6989285

[B29] Kousa O. Mullane R. Aboeata A. (2023). “Cardiorenal syndrome,” in StatPearls (Treasure Island (FL): StatPearls Publishing). 31194445

[B30] Kumar U. Wettersten N. Garimella P. S. (2019). Cardiorenal syndrome: Pathophysiology. Cardiol. Clin. 37 (3), 251–265. 10.1016/j.ccl.2019.04.001 31279419 PMC6658134

[B31] Laskowski A. Woodman O. L. Cao A. H. Drummond G. R. Marshall T. Kaye D. M. (2006). Antioxidant actions contribute to the antihypertrophic effects of atrial natriuretic peptide in neonatal rat cardiomyocytes. Cardiovasc Res. 72 (1), 112–123. 10.1016/j.cardiores.2006.07.006 16890211

[B32] Levey A. S. Eckardt K. U. Dorman N. M. Christiansen S. L. Hoorn E. J. Ingelfinger J. R. (2020). Nomenclature for kidney function and disease: Report of a kidney disease: Improving global outcomes (KDIGO) consensus conference. Kidney Int. 97 (6), 1117–1129. 10.1016/j.kint.2020.02.010 32409237

[B33] Li Y. Kang L. Rong K. Zhang Y. Suo Y. Yuan M. (2021). Renal protective effects and mechanisms of the angiotensin receptor-neprilysin inhibitor LCZ696 in mice with cardiorenal syndrome. Life Sci. 280, 119692. 10.1016/j.lfs.2021.119692 34102189

[B34] Lin L. M. Wu Y. Wu M. F. Lin J. X. (2016). Focus on the novel cardiovascular drug LZC696: From evidence to clinical consideration. Cardiovasc Drugs Ther. 30 (6), 623–633. 10.1007/s10557-016-6699-5 27858191

[B35] Writing Committee Maddox T. M. Januzzi J. L. Jr Allen L. A. Breathett K. Butler J. Davis L. L. (2021). 2021 update to the 2017 ACC expert consensus decision pathway for optimization of heart failure treatment: Answers to 10 pivotal issues about heart failure with reduced ejection fraction: A report of the American College of Cardiology solution set oversight committee. J. Am. Coll. Cardiol. 77 (6), 772–810. 10.1016/j.jacc.2020.11.022 33446410

[B36] Martens P. Nuyens D. Rivero-Ayerza M. Van Herendael H. Vercammen J. Ceyssens W. (2019). Sacubitril/valsartan reduces ventricular arrhythmias in parallel with left ventricular reverse remodeling in heart failure with reduced ejection fraction. Clin. Res. Cardiol. official J. Ger. Cardiac Soc. 108 (10), 1074–1082. 10.1007/s00392-019-01440-y 30788621

[B37] Martin F. L. Stevens T. L. Cataliotti A. Schirger J. A. Borgeson D. D. Redfield M. M. (2005). Natriuretic and antialdosterone actions of chronic oral NEP inhibition during progressive congestive heart failure. Kidney Int. 67 (5), 1723–1730. 10.1111/j.1523-1755.2005.00269.x 15840018

[B38] Mc C. F. Lefkowitz M. P. Claggett B. Anavekar N. S. Senni M. Gori M. (2020). Angiotensin-neprilysin inhibition and renal outcomes in heart failure with preserved ejection fraction. Circulation 142 (13), 1236–1245. 10.1161/CIRCULATIONAHA.120.047643 32845715

[B39] McMurray J. J. Packer M. Desai A. S. Gong J. Lefkowitz M. P. Rizkala A. R. (2014). Angiotensin-neprilysin inhibition versus enalapril in heart failure. N. Engl. J. Med. 371 (11), 993–1004. 10.1056/NEJMoa1409077 25176015

[B40] Mueller C. McDonald K. de Boer R. A. Maisel A. Cleland J. Kozhuharov N. (2019). Heart Failure Association of the European Society of Cardiology practical guidance on the use of natriuretic peptide concentrations. Eur. J. heart Fail. 21 (6), 715–731. 10.1002/ejhf.1494 31222929

[B41] Mullens W. Damman K. Testani J. M. Martens P. Mueller C. Lassus J. (2020). Evaluation of kidney function throughout the heart failure trajectory - a position statement from the Heart Failure Association of the European Society of Cardiology. Eur. J. Heart Fail 22 (4), 584–603. 10.1002/ejhf.1697 31908120

[B42] Muñoz-Durango N. Fuentes C. A. Castillo A. E. González-Gómez L. M. Vecchiola A. Fardella C. E. (2016). Role of the renin-angiotensin-aldosterone system beyond blood pressure regulation: Molecular and cellular mechanisms involved in end-organ damage during arterial hypertension. Int. J. Mol. Sci. 17 (7), 797. 10.3390/ijms17070797 27347925 PMC4964362

[B43] Myhre P. L. Vaduganathan M. Claggett B. Packer M. Desai A. S. Rouleau J. L. (2019). B-type natriuretic peptide during treatment with sacubitril/valsartan: The PARADIGM-HF trial. J. Am. Coll. Cardiol. 73 (11), 1264–1272. 10.1016/j.jacc.2019.01.018 30846338 PMC7955687

[B44] Nakagawa Y. Nishikimi T. Kuwahara K. (2019). Atrial and brain natriuretic peptides: Hormones secreted from the heart. Peptides 111, 18–25. 10.1016/j.peptides.2018.05.012 29859763

[B45] Oatmen K. E. Zile M. R. Burnett J. C. Jr Spinale F. G. (2018). Bioactive signaling in next-generation pharmacotherapies for heart failure: A review. JAMA Cardiol. 3 (12), 1232–1243. 10.1001/jamacardio.2018.3789 30484834

[B46] Owens A. T. Brozena S. Jessup M. (2017). Neprilysin inhibitors: Emerging therapy for heart failure. Annu. Rev. Med. 68, 41–49. 10.1146/annurev-med-052915-015509 27686019

[B47] Packer M. Claggett B. Lefkowitz M. P. McMurray J. J. V. Rouleau J. L. Solomon S. D. (2018). Effect of neprilysin inhibition on renal function in patients with type 2 diabetes and chronic heart failure who are receiving target doses of inhibitors of the renin-angiotensin system: A secondary analysis of the PARADIGM-HF trial. Lancet Diabetes Endocrinol. 6 (7), 547–554. 10.1016/S2213-8587(18)30100-1 29661699

[B48] Pacurari M. Kafoury R. Tchounwou P. B. Ndebele K. (2014). The Renin-Angiotensin-aldosterone system in vascular inflammation and remodeling. Int. J. Inflam. 2014, 689360. 10.1155/2014/689360 24804145 PMC3997861

[B49] Peng S. Lu X. F. Qi Y. D. Li J. Xu J. Yuan T. Y. (2020). LCZ696 ameliorates oxidative stress and pressure overload-induced pathological cardiac remodeling by regulating the sirt3/MnSOD pathway. Oxid. Med. Cell Longev. 2020, 9815039. 10.1155/2020/9815039 33014281 PMC7519988

[B50] Polhemus D. J. Trivedi R. K. Gao J. Li Z. Scarborough A. L. Goodchild T. T. (2017). Renal sympathetic denervation protects the failing heart via inhibition of neprilysin activity in the kidney. J. Am. Coll. Cardiol. 70 (17), 2139–2153. 10.1016/j.jacc.2017.08.056 29050562

[B51] Polina I. Domondon M. Fox R. Sudarikova A. V. Troncoso M. Vasileva V. Y. (2020). Differential effects of low-dose sacubitril and/or valsartan on renal disease in salt-sensitive hypertension. Am. J. Physiol. Ren. Physiol. 319 (1), F63–F75. 10.1152/ajprenal.00125.2020 PMC7468826 32463726

[B52] Potter L. R. Yoder A. R. Flora D. R. Antos L. K. Dickey D. M. (2009). Natriuretic peptides: Their structures, receptors, physiologic functions and therapeutic applications. Handb. Exp. Pharmacol. (191), 341–366. 10.1007/978-3-540-68964-5_15 19089336 PMC4855512

[B53] Romero C. A. Orias M. Weir M. R. (2015). Novel RAAS agonists and antagonists: Clinical applications and controversies. Nat. Rev. Endocrinol. 11 (4), 242–252. 10.1038/nrendo.2015.6 25666495 PMC7097622

[B54] Ronco C. Di Lullo L. (2014). Cardiorenal syndrome. Heart Fail. Clin. 10 (2), 251–280. 10.1016/j.hfc.2013.12.003 24656104

[B55] Ronco C. Haapio M. House A. A. Anavekar N. Bellomo R. (2008). Cardiorenal syndrome. J. Am. Coll. Cardiol. 52 (19), 1527–1539. 10.1016/j.jacc.2008.07.051 19007588

[B56] Ruiz-Hurtado G. Ruilope L. M. (2015). Cardiorenal protection during chronic renin-angiotensin-aldosterone system suppression: Evidences and caveats. Eur. Heart J. Cardiovasc Pharmacother. 1 (2), 126–131. 10.1093/ehjcvp/pvu023 27533982

[B57] Sabbah H. N. Zhang K. Gupta R. C. Xu J. Singh-Gupta V. (2020). Effects of angiotensin-neprilysin inhibition in canines with experimentally induced cardiorenal syndrome. J. cardiac Fail. 26 (11), 987–997. 10.1016/j.cardfail.2020.08.009 PMC7704862 32841710

[B58] Sankhe R. Rathi E. Manandhar S. Kumar A. Pai S. Kini S. G. (2021). Repurposing of existing FDA approved drugs for Neprilysin inhibition: An *in-silico* study. J. Mol. Struct. 1224, 129073. 10.1016/j.molstruc.2020.129073 32834116 PMC7422802

[B59] Savira F. Magaye R. Liew D. Reid C. Kelly D. J. Kompa A. R. (2020). Cardiorenal syndrome: Multi-organ dysfunction involving the heart, kidney and vasculature. Br. J. Pharmacol. 177 (13), 2906–2922. 10.1111/bph.15065 32250449 PMC7280015

[B60] Sible A. M. Nawarskas J. J. Alajajian D. Anderson J. R. (2016). Sacubitril/valsartan: A novel cardiovascular combination agent. Cardiol. Rev. 24 (1), 41–47. 10.1097/CRD.0000000000000093 26466333

[B61] Simões E Silva A. C. Teixeira M. M. (2016). ACE inhibition, ACE2 and angiotensin-(1-7) axis in kidney and cardiac inflammation and fibrosis. Pharmacol. Res. 107, 154–162. 10.1016/j.phrs.2016.03.018 26995300

[B62] Singh J. Burrell L. M. Cherif M. Squire I. B. Clark A. L. Lang C. C. (2017). Sacubitril/valsartan: Beyond natriuretic peptides. Heart 103 (20), 1569–1577. 10.1136/heartjnl-2017-311295 28689178

[B63] Singhania N. Bansal S. Mohandas S. Nimmatoori D. P. Ejaz A. A. Singhania G. (2020). Role of renin-angiotensin-aldosterone system inhibitors in heart failure and chronic kidney disease. Drugs context 9, 1–3. 10.7573/dic.2020-7-3 PMC7673621 33240389

[B64] Spannella F. Marini M. Giulietti F. Rosettani G. Francioni M. Perna G. P. (2019). Renal effects of sacubitril/valsartan in heart failure with reduced ejection fraction: A real life 1-year follow-up study. Intern Emerg. Med. 14 (8), 1287–1297. 10.1007/s11739-019-02111-6 31147823 PMC6853858

[B65] Suematsu Y. Jing W. Nunes A. Kashyap M. L. Khazaeli M. Vaziri N. D. (2018). LCZ696 (Sacubitril/Valsartan), an angiotensin-receptor neprilysin inhibitor, attenuates cardiac hypertrophy, fibrosis, and vasculopathy in a rat model of chronic kidney disease. J. Card. Fail 24 (4), 266–275. 10.1016/j.cardfail.2017.12.010 29325796

[B66] Thind G. S. Loehrke M. Wilt J. L. (2018). Acute cardiorenal syndrome: Mechanisms and clinical implications. Cleve Clin. J. Med. 85 (3), 231–239. 10.3949/ccjm.85a.17019 29522391

[B67] Trivedi R. K. Polhemus D. J. Li Z. Yoo D. Koiwaya H. Scarborough A. (2018). Combined angiotensin receptor-neprilysin inhibitors improve cardiac and vascular function via increased NO bioavailability in heart failure. J. Am. Heart Assoc. 7, e008268. 10.1161/JAHA.117.008268 29502102 PMC5866338

[B68] Vardeny O. Claggett B. Packer M. Zile M. R. Rouleau J. Swedberg K. (2016). Efficacy of sacubitril/valsartan vs. enalapril at lower than target doses in heart failure with reduced ejection fraction: The PARADIGM-HF trial. Eur. J. heart Fail. 18 (10), 1228–1234. 10.1002/ejhf.580 27283779 PMC5095784

[B69] Vardeny O. Tacheny T. Solomon S. D. (2013). First-in-class angiotensin receptor neprilysin inhibitor in heart failure. Clin. Pharmacol. Ther. 94 (4), 445–448. 10.1038/clpt.2013.146 23872864

[B70] Vejakama P. Ingsathit A. McKay G. J. Maxwell A. P. McEvoy M. Attia J. (2017). Treatment effects of renin-angiotensin aldosterone system blockade on kidney failure and mortality in chronic kidney disease patients. BMC Nephrol. 18 (1), 342. 10.1186/s12882-017-0753-9 29187194 PMC5706339

[B71] Vellaichamy E. Khurana M. L. Fink J. Pandey K. N. (2005). Involvement of the NF-kappa B/matrix metalloproteinase pathway in cardiac fibrosis of mice lacking guanylyl cyclase/natriuretic peptide receptor A. J. Biol. Chem. 280 (19), 19230–19242. 10.1074/jbc.M411373200 15710627

[B72] Vindhyal M. R. Khayyat S. Shaaban A. Duran B. A. Kallail K. J. (2018). Decreased renal function is associated with heart failure readmissions. Cureus 10 (8), e3122. 10.7759/cureus.3122 30338197 PMC6177062

[B73] Virzì G. M. Clementi A. de Cal M. Brocca A. Day S. Pastori S. (2015). Oxidative stress: Dual pathway induction in cardiorenal syndrome type 1 pathogenesis. Oxid. Med. Cell Longev. 2015, 391790. 10.1155/2015/391790 25821554 PMC4364374

[B74] Volpe M. (2014). Natriuretic peptides and cardio-renal disease. Int. J. Cardiol. 176 (3), 630–639. 10.1016/j.ijcard.2014.08.032 25213572

[B75] Voors A. A. Gori M. Liu L. C. Y. Claggett B. Zile M. R. Pieske B. (2015). Renal effects of the angiotensin receptor neprilysin inhibitor LCZ696 in patients with heart failure and preserved ejection fraction. Eur. J. Heart Fail 17 (5), 510–517. 10.1002/ejhf.232 25657064

[B76] Yang C. C. Chen Y. T. Chen C. H. Li Y. C. Shao P. L. Huang T. H. (2019). The therapeutic impact of entresto on protecting against cardiorenal syndrome-associated renal damage in rats on high protein diet. Biomed. Pharmacother. = Biomedecine Pharmacother. 116, 108954. 10.1016/j.biopha.2019.108954 31108352

[B77] Yuan C. Wang Y. Su W. Jia S. (2010). Research progress of cardiorenal syndrome. Med. Rev. 16 (09), 1404–1407.

[B78] Zhang J. Li M. Yang Y. Yan Y. Li J. Qu J. (2015). NPR-A: A therapeutic target in inflammation and cancer. Crit. Rev. Eukaryot. Gene Expr. 25 (1), 41–46. 10.1615/critreveukaryotgeneexpr.2015012447 25955817

[B79] Ziff O. J. Covic A. Goldsmith D. (2016). Calibrating the impact of dual RAAS blockade on the heart and the kidney - balancing risks and benefits. Int. J. Clin. Pract. 70 (7), 537–553. 10.1111/ijcp.12803 27278080

